# Human Papillomavirus Infection: Knowledge, Attitudes, and Behaviors among Lesbian, Gay Men, and Bisexual in Italy

**DOI:** 10.1371/journal.pone.0042856

**Published:** 2012-08-08

**Authors:** Concetta P. Pelullo, Gabriella Di Giuseppe, Italo F. Angelillo

**Affiliations:** Department of Experimental Medicine, Second University of Naples, Naples, Italy; IPO, Inst Port Oncology, Portugal

## Abstract

**Background:**

This cross-sectional study assess knowledge, attitudes, and behavior towards the human papillomavirus (HPV) and the vaccination among a random sample of 1000 lesbian, gay men, and bisexual women and men.

**Methods:**

A face-to-face interview sought information about: socio-demographic characteristics, knowledge about HPV infection, perception of risk towards HPV infection and/or cervical, anal, and oropharyngeal cancers, perception of the benefits of a vaccination to prevent cervical, anal, and oropharyngeal cancers, sexual behaviors, health-promoting behaviors, and willingness to receive the HPV vaccine.

**Results:**

Only 60.6% had heard about the HPV infection and this knowledge was significantly higher in female, in those being a member of a homosexual association, in those having had the first sexual experience at a younger age, in those having received information about the HPV infection from physicians, and in those having no need of information about HPV infection. A higher perceived risk of contracting HPV infection has been observed in those younger, lesbian and gay men, who have heard of HPV infection and knew the risk factors and its related diseases, who have received information about HPV infection from physicians, and who need information about HPV infection. Only 1.7% have undergone HPV immunization and 73.3% professed intent to obtain it in the future. The significant predictors of the willingness to receive this vaccine were belief that the vaccination is useful, perception to be at higher risk of contracting HPV infection, and perception to be at higher risk of developing cervical, anal, and oropharyngeal cancers.

**Conclusions:**

Information and interventions are strongly needed in order to overcome the lack of knowledge about HPV infection and its vaccination. Inclusion of boys in the national vaccination program and initiate a catch-up program for men who have sex with men up to 26 years may reduce their burden of HPV-related disease.

## Introduction

Genital human papillomavirus (HPV) is one of the most prevalent sexually transmitted infection in several countries worldwide. High-risk HPV genotypes cause virtually all cases of cervical cancer, and also may lead significant health complications in men including anal and penile cancers, genital warts and oropharyngeal cancers, whereas low-risk genotypes can cause genital warts and respiratory papillomatosis [1], [2]. HPV infection is highly prevalent among the sexual active population, specially the one between 16–25 years old, and often it occurs soon after sexual debut or the introduction of new sexual partners [1], [3]. Moreover, HPV-related disease is particularly common among those with certain high-risk behaviors, including women who have sex with women, men who have sex with men (MSM), and bisexual women and men [4], [5].

To reduce the chances of getting HPV infection and its related diseases, people should use condoms during every sex act, limit their number of sexual partners, and choose a partner who has had no or few prior sexual partners [1]. However, campaigns for use of condoms, sexual abstinence and limitations in number of sexual partners have low efficacy [6]–[9]. The use of condom does not eliminate the risk of HPV-infection because HPV can be transmitted from skin not covered by the condom. The HPV vaccine gives better protection of HPV-infection than condoms, but a combination of both vaccine and condom provides the greatest protection [10]. HPV vaccination has given hope for reducing the incidence of infections, the morbidity and the mortality of the HPV-related cancer. The most recent recommendations include the administration of the vaccine to adolescents and young adult females aged 9 through 26 years and also for eligible males of similar ages in order to reduce their likelihood of acquiring genital warts and to prevent cancer [5]. Moreover, HPV vaccination of men who have sex with men has been indicated to be a cost-effective intervention for the prevention of genital warts and anal cancer [11]. MSM have increased risk of acquiring HPV-infection and HPV-related diseases than men who have sex with women [12]–[15]. MSM often have multiple sexual partners and practice anal and oral sex [16]. Even though anal cancer is more prevalent in female than males, the risk of anal cancer in MSM is higher [15]. MSM are also at increased risk of developing cancers in sites that are associated with HPV in the oral cavity and the oropharynx compared with other men [17], [18]. There have been an increase in oropharyngeal cancer the last 20 years and this is probably due to increasing number of partners and change in sexual practice [3].

It is important to investigate whether different groups of population have adequate knowledge and perceive their behavior as risky for the acquisition of HPV in order to provide appropriate and useful information to them. Although there is a large body of research regarding knowledge, willingness to accept for themselves or to provide HPV vaccination, and behavior regarding HPV among adolescents [19], [20], parents [21]–[23], and health care providers [24]–[27], there has been a paucity of information on lesbian, gay men, and bisexual women and men [28]–[34]. Therefore, one of the purposes of the present study was to address these limitations by recruiting a large sample of lesbian, gay men, and bisexual women and men in Italy in the hope of obtaining results which would accurately reflect the knowledge, attitudes, and behavior of this population towards the HPV and the vaccination. A further aim was to examine the factors that are related to knowledge, attitudes, and behavior in this population.

## Materials and Methods

The present study employed a cross-sectional design and a large random sample of 1152 lesbian, gay men, and bisexual women and men was recruited during the period from March through June 2011 in the city of Naples, South of Italy, from nineteen public meeting places as bars, pubs, and clubs randomly selected from the list of places habitually frequented by the lesbian, gay men, and bisexual women and men population.

Sample size was calculated before study initiation, considering a prevalence of 65% of lesbian, gay men, and bisexual women and men who have heard about HPV, using a confidence interval of 95%, a margin of error of 5%, and a design effect of 2. Finally, considering a response rate of 70%, a total sample of 1000 lesbian, gay men, and bisexual women and men individuals was sought. During each sampling event, one staffer counted the numbers of individuals entering and every 10-th person was selected for inclusion in the sample population.

The study tool was a questionnaire administered by a face-to-face interview conducted by six lesbian, gay men, and bisexual women and men interviewers' who have been well-trained prior to data collection. The interviewers approached the lesbian, gay men, and bisexual women and men in each public venue and were given verbally information on the purpose of the study, emphasized that the participation in the study was voluntary so that no one would feel obliged to participate, anonymous, that given answers were confidential and will only be analyzed as aggregates, and without incentive for questionnaire completion. Consent was implied if they choose to complete the questionnaire.

The survey questionnaire sought data relating to socio-demographic characteristics, knowledge about HPV infection, perception of risk towards HPV infection and/or cervical, anal, and oropharyngeal cancers, perception of the benefits of a vaccination to prevent cervical, anal, and oropharyngeal cancers, sexual behaviors, health-promoting behaviors, willingness to receive the HPV vaccine, and sources of and needs of information regarding HPV infection and vaccine. The questions were constructed based mainly on previously used in similar surveys questionnaires [19], [35], [36].

The respondents' knowledge was tested with seven items relating to HPV infection and its vaccination, which required yes, no or do not know responses or multiple-choice questions. Respondents' attitude were asked to indicate their perception of risk towards HPV infection and/or cervical, anal, and oropharyngeal cancers on a 10-point Likert scale ranging from 1 (not worried) to 10 (extremely worried); their opinion about the perceived benefits of a vaccination to prevent cervical, anal, and oropharyngeal cancers on a 10-point Likert scale, with higher score indicating a more positive attitude towards the HPV vaccine; their willingness to receive HPV vaccine was also asked on a 10-point Likert scale ranging from 1 to 10, with a higher score indicating a greater intention to get vaccinated. Their beliefs about HPV infection were measured on a 3-point Likert-type scale with options agree, uncertain, and disagree; and their opinion whether female and/or male should be vaccinated. Three closed-questions and four open-questions assessed the sexual behaviors. Respondents were asked to indicate whether or not: (1) they have ever had sexual intercourse and age at first sexual intercourse; (2) lifetime gender of sexual partner(s); (3) number and gender of sexual partner(s) in the past year; (4) each alter identified as a sexual partner was regular (defined as “person with whom the participant had sex and to whom he/she felt most committed”), and casual partner (defined as “person with whom the participant had sex but to whom he/she did not feel committed”) in the past year; and (5) use of condom during sexual intercourse with regular(s) and casual partner(s) in the past year measured on a five-point Likert scale ranging from ‘never’ to ‘every time’. With regard to health-promoting behavior, respondents were asked to indicate whether or not: (1) they have received health checkup in the past year and the reason(s); (2) the physician discussed about HPV vaccine; and (3) they had been vaccinated. The questions regarding the sources of information on HPV infection and vaccine were asked by including a list of options with the possibility to indicate more than one source, whereas the question about educational needs included a response in the ‘yes’ and ‘no’ format. Additionally the following respondent data were collected: gender, age, sexual orientation, education level, marital status, number of sons, occupation, whether they were living alone or not, and whether they were members of a homosexual association (defined as an organization which promotes activities on education and prevention and fights against violence and discrimination).

The questionnaire was reviewed for content, readability, and comprehensiveness by a sample of 50 lesbian, gay men, and bisexual. A group of experts reviewed the format and content of the items, as well as the content validity of the instrument as a whole. The internal reliability was assessed using Cronbach's α.

Before the study initiation, the approval of the Ethics Committee of the Second University of Naples was requested and obtained.

### Statistical analysis

There were three primary outcomes of interest in this study: a) have heard that HPV is a sexually transmitted infection (no = 0; yes = 1), (Model 1); b) perception of risk of contracting HPV infection (continuous), (Model 2); and c) willingness to receive the HPV vaccine (no = 0; yes = 1), (Model 3). In all models, the following independent variables were included: gender (male = 0; female = 1), age (continuous, in years), education level (three categories: middle school or lower = 1; high school = 2; college degree or higher = 3), sexual orientation (three categories: lesbian = 1; gay men = 2; bisexual = 3), occupation (unemployed = 0; employed = 1), age at first sexual intercourse (continuous, in years), number of casual partners in the last year (continuous), and at least a health checkup in the last year (no = 0; yes = 1). The following variables were also included: member of an homosexual association (no = 0; yes = 1) in Model 1; physician as source of information about HPV infection (no = 0; yes = 1), and need of additional information about HPV infection (no = 0; yes = 1) in Models 1 and 2; number of regular partners in the last year (continuous) in Models 1 and 3; have heard that HPV is a sexually transmitted infection, know that it can cause cervical, anal, and oropharyngeal cancers, that multiple sexual partners of the opposite gender and/or same gender and condom failure increase the risk of contracting the HPV infection (no = 0; yes = 1) in Models 2 and 3; have heard about HPV vaccine and know the preventive measures (condom use in sexual intercourse, reduction of the number of sexual partners, vaccination) and that the diseases may be prevented by the vaccination (cervical, anal, and oropharyngeal cancers) (no = 0; yes = 1), perception of risk of contracting HPV infection (continuous), perception of risk of developing cervical, anal, and oropharyngeal cancers (continuous), perceived benefits of a vaccination to prevent cervical, anal, and oropharyngeal cancers (continuous), physician as source of information about HPV infection and vaccine (no = 0; yes = 1), and need of additional information about HPV infection and vaccine (no = 0; yes = 1) in Model 3.

1. Bivariate appropriate tests (t-tests and chi-square tests) have been used to assess the univariate associations between each of the independent characteristics and the different outcomes of interest. After performing the exploratory bivariate analyses, only those variables found to be associated with the outcomes of interest at the *p*-value ≤0.25 level were subsequently introduced into a multivariate regression model to assess their independent contribution to predict each outcome. Then, logistic and linear regressions have been performed to model multivariable associations between the independent characteristics and respectively the dichotomous and continuous outcomes of interest. Backward stepwise procedures were applied so that the final models only included characteristics providing a significant explanation of outcomes, in which the criterion for entering and being retained in the model has a *p*-value respectively of 0.2 and 0.4. Adjusted odds ratios (ORs) and 95% confidence intervals (CIs) were presented in the logistic regression models. Standardized regression coefficients (β) were presented in the linear regression models. All statistical tests were two-sided and the level of statistical significance was set at 0.05 throughout the whole study. Analyses were performed using Stata 10 software (StataCorp. 2007. Stata Statistical Software: Release 10. College Station, TX: StataCorp LP).

## Results

In terms of reliability of the instrument, the Cronbach's α reliability coefficients indicated a high internal consistency. Calculation of the content validity indicated the unanimous agreement with the questionnaire's content and clarity.

Of the 1152 lesbian, gay men, and bisexual women and men that were approached, a total of 1000 agreed to participate for an effective response rate of 86.8%. The distribution of the surveyed participants according to socio-demographic characteristics by sexual orientation are summarized in Table 1. Almost two-thirds of the survey respondents were male, the mean age was 26.6 years, more than half self-identified as gay men, the vast majority was not married, and had a high school education or higher.

**Table 1:** Selected characteristics of the study population according to the sexual orientation.

	Gay men (N = 566)	Lesbians (N = 279)		Bisexual men (N = 64)	Bisexual women (N = 91)	
*Characteristic*	N (%)	N (%)	*p*-value	N (%)	N (%)	*p*-value
*Age (years)*	26.7±7.4(17–62)*	26.1±5.4(17–64)*		26.1±6.4(16–51)*	27.4±7.1(17–68)*	
*Marital status*						
Married	512 (90.5)	223 (79.9)	<0.0001	48 (75)	71 (78)	
Other	54 (9.5)	56 (20.1)		16 (25)	20 (22)	
*Education level* a						
Middle school or less	76 (13.5)	19 (6.8)		10 (15.6)	11 (12.1)	
High school	407 (72)	180 (64.5)	<0.0001	46 (71.9)	38 (41)	0.001
College degree or higher	82 (14.5)	80 (28.7)		8 (12.5)	42 (46.1)	
*Occupation* a						
Employed	505 (89.2)	252 (90.3)		55 (87.3)	80 (87.9)	
Unemployed	61 (10.8)	27 (9.7)		8 (12.7)	11 (12.1)	

* Mean±Standard deviation (Range).

a The respondents are 999.

Table 2 provides an overview of the answers to each question regarding the knowledge according to sexual orientation. When we asked the participants the different true or false questions about the risk factors, HPV related diseases, the preventive measures, and the diseases prevented by the vaccination, the frequencies of those who answered them correctly varied considerably. Indeed, the vast majority knew that unprotected sex was the main risk factor and that the condom use was a preventive measure, whereas other risk factors were less frequently acknowledged. Of note, less than half (42.1%) of the respondents reported that they had heard about the HPV vaccine and 85% of those who had heard of the vaccination prior to the survey knew that cervical cancer can be prevented by vaccination. Overall, only 60.6% participants reported that they had heard about the HPV infection and a multivariate logistic regression analysis was conducted to explore whether the respondents with various characteristics mastered different levels of knowledge. The results of the logistic regression analysis revealed that the following factors were statistically significantly associated with a higher level of knowledge: female gender (OR = 2.62; 95% CI 1.42–4.84), being a member of an homosexual association (OR = 2.5; 95% CI 1.68–3.73), having had the first sexual experience at a younger age (OR = 0.9; 95% CI 0.85–0.96), having received information about the HPV infection from physicians (OR = 12.32; 95% CI 5.85–25.94), and having no need of additional information about the HPV infection (OR = 0.34; 95% CI 0.23–0.52). The education has also an impact on the level of knowledge since respondents with a middle school or lower (OR = 0.25; 95% CI 0.14–0.46) and high school (OR = 0.51; 95% CI 0.33–0.77) were less knowledgeable compared to those with a college degree or higher (Model 1 in Table 3).

**Table 2:** Knowledge about Human Papillomavirus (HPV) of the study population according to the sexual orientation.

	Gay men (N = 566)	Lesbians (N = 279)		Bisexual men (N = 64)	Bisexual women (N = 91)	
	N (%)	N (%)	*p*-value	N (%)	N (%)	*p*-value
*Have heard about HPV infection*						
Yes	309 (54.6)	183 (65.6)	0.002	34 (53.1)	70 (76.9)	0.002
No	257 (45.4)	96 (34.4)		30 (46.9)	21 (23.1)	
*Risk factors* *						
Condom failure (true)	493 (87.1)	241 (86.4)		58 (90.6)	72 (79.1)	
Unprotected sex with partners of the same gender (true)	466 (82.3)	247 (88.5)	0.02	52 (81.2)	70 (76.9)	
Unprotected sex with partners of the opposite gender (true)	453 (80)	254 (91)	<0.0001	54 (84.4)	71 (78)	
Multiple sexual partners of the opposite gender (true)	172 (30.4)	87 (31.2)		14 (21.9)	26 (28.6)	
Multiple sexual partners of the same gender (true)	179 (31.6)	73 (26.2)		14 (21.9)	27 (29.7)	
Sexual intercourse at an early age (true)	107 (18.9)	45 (16.1)		14 (21.9)	14 (15.4)	
*HPV related diseases* *						
Cervical cancer (true)	316 (55.8)	187 (67)	0.002	33 (51.6)	67 (73.6)	0.005
Anal cancer (true)	301 (53.2)	158 (56.6)		31 (48.4)	42 (46.2)	
Oral cancer (true)	266 (47)	124 (44.4)		20 (31.3)	32 (35.2)	
Urinary infection (false)	151 (26.7)	65 (23.3)		16 (25)	22 (24.2)	
Bowel cancer (false)	91 (16.1)	16 (5.7)	<0.0001	5 (7.8)	2 (2.2)	
Lung cancer (false)	35 (6.2)	4 (1.4)	0.001	3 (4.7)	0 (-)	0.04
*Preventive measures for HPV infection* *						
Condom use in sexual intercourse (true)	488 (86.2)	241 (86.4)		57 (89.1)	72 (79.1)	
Vaccination (true)	360 (63.6)	160 (57.4)		43 (67.2)	47 (51.7)	
Oral contraceptive (false)	248 (43.8)	103 (36.9)		13 (20.3)	26 (28.6)	
Pap test (false)	215 (38)	61 (21.9)	<0.0001	22 (34.4)	13 (14.3)	0.003
Reducing the number of same gender partners (true)	170 (30)	65 (23.3)	0.04	13 (20.3)	17 (18.7)	
Reducing the number of partners of the opposite gender (true)	158 (27.9)	71 (25.5)		14 (21.9)	20 (22)	
Late start of sexual activity (true)	124 (21.9)	53 (19)		11 (17.2)	9 (9.9)	
*Have heard about HPV vaccine*						
Yes	238 (42.1)	112 (40.1)		19 (29.7)	52 (57.1)	0.001
No	328 (57.9)	167 (59.9)		45 (70.3)	39 (42.9)	
*HPV vaccine prevent* * a						
Cervical cancer (true)	194 (34.3)	104 (37.3)		13 (20.3)	47 (51.7)	<0.0001
Anal cancer (true)	169 (29.9)	72 (25.8)		7 (10.9)	24 (26.4)	0.02
Oral cancer (true)	144 (25.4)	55 (19.7)		5 (7.8)	15 (16.5)	
Urinary infection (false)	82 (14.5)	14 (5)	<0.0001	6 (9.4)	6 (6.6)	
Bowel cancer (false)	48 (6.5)	5 (1.8)	0.001	0 (-)	1 (1.1)	
Lung cancer (true)	22 (3.9)	2 (0.7)	0.007	1 (1.6)	0 (-)	

* Respondents could have selected more than one response.

a Only for those who reported that they have heard about HPV vaccine.

**Table 3:** Multivariate logistic (Models 1 and 3) and linear regression (Model 2) analysis to characterize factors associated with the different outcomes of interest.

Variable	OR	SE	95% CI	*p*-value
**Model 1.** Have heard that HPV is a sexually transmitted infection (Sample size = 924)				
Log likelihood = −507.52, χ^2^ = 224 (10 df), *p*<0.0001				
Who is a member of an homosexual association	2.5	0.51	1.68–3.73	<0.001
Education level				
Middle school or less	0.25	0.08	0.14–0.46	<0.001
High school	0.51	0.11	0.33–0.77	0.002
College degree or higher*	1.0	-	-	-
Who did not need additional information about HPV infection	0.34	0.07	0.23–0.52	<0.001
Who have received information about the HPV infection from physicians	12.32	4.68	5.85–25.94	<0.001
Who have had the first sexual intercourse at a lower age	0.9	0.03	0.85–0.96	0.001
Female gender	2.62	0.82	1.42–4.84	0.002
Sexual orientation				
Bisexual*	1.0	-	-	-
Lesbian	0.56	0.18	0.29–1.07	0.079
Who have had a lower number of casual sexual partners in the last year	0.98	0.01	0.96–1.01	0.16
Who have had a lower number of regular sexual partners in the last year	0.92	0.05	0.82–1.04	0.17
**Model 3.** Willingness to receive the HPV vaccine (Sample size = 918)				
Log likelihood = −406.39, χ^2^ = 252.34 (8 df), *p*<0.0001				
Who perceived that the vaccination was useful to prevent cervical, anal, and oropharyngeal cancers	1.47	0.06	1.36–1.6	<0.001
Who perceived to be at higher risk of contracting HPV infection	1.12	0.06	1.01–1.24	0.028
Who perceived to be at higher risk of developing cervical, anal, and oropharyngeal cancers	1.12	0.06	1.01–1.24	0.033
Sexual orientation				
Bisexual*	1.0	-	-	-
Gay men	1.64	0.39	1.03–2.62	0.037
Lesbian	1.48	0.39	0.87–2.49	0.15
Employed	1.71	0.46	1.01–2.92	0.048
Who have had a lower number of regular sexual partners in the last year	1.06	0.07	0.93–1.22	0.36
Who need additional information about HPV infection and vaccine	1.2	0.24	0.8–1.79	0.38
**Model 2.** Perception of risk of contracting HPV infection (Sample size = 926)	Coeff	SE	*t*	*p*-value
F (10,915) = 5.85, *p*<0.0001, R^2^ = 0.06%, adjusted R^2^ = 4.98%				
Who need additional information about HPV infection	0.63	0.19	3.27	0.001
Who have received information about the HPV infection from physicians	0.69	0.22	3.2	0.001
Sexual orientation				
Bisexual*				
Gay men	0.76	0.23	3.34	0.001
Lesbian	0.71	0.25	2.86	0.004
Younger age	−0.04	0.01	−2.96	0.003
Who have heard that HPV is a sexually transmitted infection and know the diseases that it can cause and its risk factors	0.94	0.37	2.52	0.012
Who have had a higher number of casual sexual partners in the last year	0.02	0.01	1.43	0.15
Who had at least a health checkup in the last year	0.2	0.17	1.18	0.24
Education level				
High school	0.2	0.17	1.15	0.25
College degree or higher*	1.0	-	-	-
Who have had first sexual intercourse at lower age	−0.26	0.03	−0.85	0.39
Constant	6.02			

* Reference category.

Respondents rated their level of agreement for three statements regarding HPV infection. Regarding perceived severity, respectively 21.4% and 47.4% considered that the infection was rare and serious and 64.4% agreed that HPV infection may be prevented. The mean value of respondents' attitude regarding the utility of the vaccination in order to prevent the developing of cervical, anal, and oropharyngeal cancers, measured on a ten-point Likert scale ranging from 1 to 10 with higher scores indicating high utility, was 6.9, with 19% reported a very favorable opinion by responding 10. The mean value of the perceived personal risk of contracting HPV infection, on a scale from 1 to 10, was 6.2. Only 10% reported that they are at risk and were concerned about contracting HPV infection by responding 10 to the question. To determine which factors were related to a participant's perceived risk of contracting HPV infection a multivariate linear regression analysis was performed and several factors have been found to be strongly linked with a higher risk perception. The regression model showed that participants were more likely to perceive a higher risk of acquire HPV infection if they were of younger age, lesbian and gay men, have heard of HPV infection and knew the risk factors and its related diseases, have received information about HPV infection from physicians, and who need additional information about HPV infection (Model 2 in Table 3).

Only 17 responders (1.7%) reported having undergone HPV immunization, the majority, 14 (82.4%) were females with a mean age of 29.5 years and 3 (17.6%) were male with a mean age of 29.3 years. The last question concerned the attitude of the subjects to the vaccination indicated that approximately three-quarters (73.3%) responded “yes” on being asked about willingness to receive HPV vaccination. A multivariate logistic regression analysis was developed to discern whether level of HPV knowledge, socio-demographics, and sexual behavior would predict respondent's vaccination intention. The results showed that were more willing to undergo future HPV vaccine gay men (OR = 1.64; 95% CI 1.03–2.62), those employed (OR = 1.71; 95% CI 1.01–2.92), those who believe that the vaccination is useful (OR = 1.47; 95% CI 1.36–1.6), who perceived to be at higher risk of contracting HPV infection (OR = 1.12; 95% CI 1.01–1.24), and who perceived to be at higher risk of developing cervical, anal, and oropharyngeal cancers (OR = 1.12; 95% CI 1.01–1.24) (Model 3 in Table 3). The most common reasons for intending to accept vaccination were reducing the risk of acquiring HPV infection (54.3%) and feeling to be at risk of getting HPV infection (37.2%), whereas the main reasons for not willing were not feeling at risk of getting HPV infection (41.8%) and concerned about vaccine efficacy (29.3%).

Some of the sexual behavior pertinent to the transmission of HPV inquired into in this study included having casual sex within the previous 12 months, having multiple sexual partners, and consistent condom use when having sexual intercourse with a casual partner. The results relative to the sexual activity and HPV vaccination are shown in Table 4. Almost all participants (94.3%) reported having been engaged in sexual intercourse and the mean age at first experience was 15.7 years. Mean number of casual sexual partners during the previous 12 months was 6.5. Less than half were not currently dating anyone. Generally, 67.9% respondents had casual sex in the previous 12 months, out of which 80.5% had two or more casual sexual partners. Data concerning condom use, showed that than one-fourth and less than half said that they “always” used condoms during sexual intercourse with current regular and casual partners, respectively, while 44.7% and 18.3% never used condoms.

**Table 4:** Reported behaviors related to sexual activity and HPV vaccination of the study population.

		N (%)
*Ever had sex*	Yes	943 (94.3)
	No	57 (5.7)
*Age at first sexual intercourse* a		15.7±2.7 (7–26)*
*Lifetime sexual partners* a	Same gender	570 (60.5)
	Both gender	368 (39)
	Opposite gender	5 (0.5)
*Sexual partners in the past year* a	Same gender	914 (97.5)
	Opposite gender	150 (39.2)
*Number of sexual partners of the same gender in the past year* a		6±7 (1–50)*
*Number of sexual partners of the opposite gender in the past year* a		4±4.2 (1–22)*
*Sexual casual partners in the past year* a b	Yes	640 (67.9)
	No	302 (32.1)
*Number of casual sexual partners in the past year* c		6.5±6.9 (1–52)*
*Condom use with casual sexual partners in the past year* c	Every time	263 (41)
	Other	377 (59)
*Have current regular sexual partner* a	Yes	494 (52.4)
	No	448 (47.6)
*Number of regular sexual partners in the past year* d		1.7±1.6 (1–16)*
*Condom use with current regular sexual partners in the past year* d	Always	118 (23.9)
	Other	376 (76.1)
*At least a health checkup in the last year* e	Yes	314 (31.5)
	No	684 (68.5)
*Physician provided information about HPV vaccine* f	Yes	44 (14)
	No	270 (86)
*Had been vaccinated* e	Yes	17 (1.7)
	No	981 (98.3)

* Mean±Standard deviation (Range).

a Only for those that they have had sexual intercourse.

b The respondents are 942.

c Only for those that they have had casual sexual partners.

d Only for those that they have had regular sexual partners.

e The respondents are 998.

f Only for those that they underwent a health checkup in the past year.

Of those 66.4% and 55.4% that had received information about HPV infection and vaccination, the most trusted sources of information was internet (53.1% and 50.8%), followed by friends and parents (45.7% and 38%), and homosexual associations (29.6% and 32.4%). Physicians were mentioned as source of information by less than one-fourth (24.4% and 23.3%) of the respondents. However, a clear majority of the overall sample (78.8% and 84.4%) reported that they were open to further information.

## Discussion

To the best of our knowledge, the present study is the largest and more detailed investigation conducted to specifically collect data on knowledge, attitudes, and behavior regarding HPV and vaccination from lesbian, gay men, and bisexual women and men in Italy and also to evaluate the factors relating to these outcomes.

The research findings revealed a non adequate level of knowledge, manifest in the responses given in all categories of questions. Indeed, this study revealed that 59.6% participants had heard about the HPV infection. In comparison with the results of other similar previous studies from different countries, this rate was better than what has been reported in Australia in a survey among gay men and other homosexually active men in which a total of 44.8% had heard of the HPV infection [28]. By contrast, a considerably higher value has been observed in three surveys conducted in the United States in samples of men who self-identified as either gay or bisexual with 79% [31], [32] and with 93% reported hearing about HPV [34]. A higher value has also been observed in women identified as lesbian and bisexual in Australia since 70.5% and 78.2% had heard of the HPV prior to their participation, respectively [29]. With regard to the level of knowledge regarding the vaccine, less than half reported that they had heard about the HPV vaccine (42.1%). This value was better than those observed in a study conducted in Australia among men who have sex with men with only 30% who were aware that there was a vaccine available for protection against infection with certain HPV types [30] and in the already mentioned survey in the United States since only 26% were aware of an HPV vaccine for males [34]. Higher values have been observed in Australia with 50% and 54.6% of lesbian and bisexual women that had heard of the HPV vaccine [29]. A considerably higher level of knowledge has been reported in the United States, in the already reported survey, since 73% had heard of HPV vaccine [32] and in a sample of HIV-positive and HIV-negative gay men with values 75% and 76%, respectively [33]. The findings suggest the urgent need for public education that explicitly addresses such knowledge deficiency among this group of subjects.

Regarding the attitudes towards HPV, less than half of the sample considered that the HPV infection was serious and two-thirds believed that it may be prevented. Another major finding was that the mean value of the perceived personal risk of contracting HPV infection was 6.2 and only 10% reported high concern. It is also worth pointing out that, since only 1.7% of the sample reported having had received the HPV vaccine, general attitudes towards vaccination were encouraging because more than two-thirds (72.1%) of unvaccinated survey participants expressed their intention to get vaccinated against HPV infection. The level of intention to get vaccinated observed in the sample was similar when compared with the values from previously conducted surveys. For example, in the Unites States approximately 74% of gay and bisexual men [32] and 74% and 78% of HIV-negative and HIV-positive gay men were willing to accept HPV vaccine [33]. The value is considerably higher than the 36% of gay and bisexual men [34] and the 39.4% and 56.6% of lesbian and bisexual women in Australia who would consider having the vaccine [29]. It should be highlighted the notable differences between the willingness to accept HPV vaccine in some of these surveys, mainly ranging from about 36% to about 56.6%, and the rate observed in the present study (72.1%). Various explanations could be considered. Among the most obvious is that such studies only documented the willingness to receive immunization, which may not necessarily reflect the actual behavior. Comparison of these results should also take into consideration the role of the timing of each study given the extensive increasing attention regarding the HPV vaccine that may have a potential impact on the level of knowledge. In the present study, the main reasons given for accept and non-accept the HPV vaccination correspond with those from previous research conducted in different countries by other authors [32]–[34]. The reasons given for vaccination can be summarized as follows: it is important to be protected against disease, the effectiveness of the vaccine, and the feeling to be at risk of contracting the HPV infection. The reasons for non-vaccination included the concept that perceived lack of effectiveness of the vaccine and fear of vaccine-related adverse reactions effects. As the majority refused HPV vaccination due to concerns about efficacy and safety of the vaccine, promotion of HPV vaccine should be supported with data about the vaccine efficacy and safety.

Using exploratory multivariate linear and logistic regression analyses, a few factors were associated with the different outcomes of interest, and most were in the direction that would be expected. The finding that physician as source of information about HPV infection was a key determinant of HPV knowledge it is not surprising and it is consistent with the literature on other vaccines and supports the importance of the role of the physician regarding the acceptability of the HPV vaccine. Another finding of the current study was that among the socio-demographic variables analyzed, gender and level of education were the only two found to be important predictors of HPV knowledge. Specifically, females and respondents with a university or a baccalaureate degree were most knowledgeable than male and than those with a lower level of education. Of particular interest the finding that the age at first sexual intercourse was also a significant predictor of the knowledge, with a higher level among those who have had their first experience at a younger age. The analysis also revealed that respondents who reported greater worry about getting HPV infection, about getting cervical, anal, and oropharyngeal cancers, and who perceived higher utility of the HPV vaccination were more willing to undergo in the future this vaccination. Finally, lesbian, gay men, and bisexual differed in their beliefs related to HPV infection. For example, lesbian and gay men were more likely than were bisexual to perceive a higher risk of acquire HPV infection and they were also more willing, although not significant, to undergo HPV vaccination than were bisexual. Interventions to increase HPV vaccination may need to be tailored to differing beliefs of lesbian, gay men, and bisexual. It should be noted that the vaccines are licensed for people through age 26 years and those who are sexually active may also benefit from the vaccine.

To appreciate the results of this study, consideration should be given to potential methodological limitations. First, the survey relied on a face-to-face interview data and some participants might feel uncomfortable with some sensitive questions such as those about sexual behaviors, and this may have limited honest responses with the risk that respondents could give a more positive picture of their engagement than would be revealed by other data collection methods. Also the positive response towards HPV vaccination could be explained because participants gave the answer they thought would be the most socially desirable, instead of giving the answer they truly wanted to give. Both problems have been limited by the facts that the interviewers were lesbian, gay men, and bisexual women and men, which may have helped build rapport in the interviewee-interviewer relationship and may have meant respondents found not difficult to discuss certain subjects matter explored in the questionnaire. Moreover, it has been guaranteed to the respondents that the method of data collection had no identifiers thus ensuring confidentiality and anonymity. Second, self-report bias, as in surveys asking questions about sexual behaviors, because participants may have responded in a way in order to make them look as good as possible and, therefore, is possible, for example, an underestimation of the number of casual partners. Third, this survey represents cross-sectional information and therefore allows to only observe associations and not attribution of cause and effect due to temporal relationship between the predictor variables and the different outcomes of testing. Even with those potential limitations, however, this research involved a well-designed survey and the broad range of public meeting places habitually frequented by a hard-to-reach population of lesbian, gay men, and bisexual women and men population, the random sampling, together with the response rate on the questionnaire that was very high strengthen the accurate and valuable results and indicates that responses are representative of all people approached to participate.

In conclusion, this study provides important insights into the beliefs and experiences of lesbian, gay men, and bisexual women and men regarding HPV infection and vaccination from which decisions are recommended. Health promotion programs are not provided for this selected population and, therefore, the study supports the need to disseminate accurate information and interventions, including continuous health promotion and education, by taking into account the identified areas of misperceptions in order to overcome the lack of knowledge about HPV infection and its vaccination. Inclusion of boys in the national vaccination program and initiate a catch-up program up to 26 years for MSM may reduce their burden of HPV-related disease.
